# The financial sustainability of the World Health Organization and the political economy of global health governance: a review of funding proposals

**DOI:** 10.1186/s12992-018-0436-8

**Published:** 2018-11-29

**Authors:** Srikanth K. Reddy, Sumaira Mazhar, Raphael Lencucha

**Affiliations:** 1 Faculty of Medicine, School of Physical and Occupational Therapy, McGill University, Rabinovitch House, 3640 de la Montagne, Montreal, QC H3G 2A8 Canada; 2 McGill University Health Centre, McGill University, Montreal, Canada

**Keywords:** World Health Organization, Governance for health, Financing, International political, Economy, Reform

## Abstract

The World Health Organization (WHO) continues to experience immense financial stress. The precarious financial situation of the WHO has given rise to extensive dialogue and debate. This dialogue has generated diverse technical proposals to remedy the financial woes of the WHO and is intimately tied to existential questions about the future of the WHO in global health governance. In this paper, we review, categorize, and synthesize the proposals for financial reform of the WHO. It appears that less contentious issues, such as convening financing dialogue and establishing a health emergency programme, received consensus from member states. However, member states are reluctant to increase the assessed annual contributions to the WHO, which weakens the prospect for greater autonomy for the organisation. The WHO remains largely supported by earmarked voluntary contributions from states and non-state actors. We argue that while financial reform requires institutional changes to enhance transparency, accountability and efficiency, it is also deeply tied to the political economy of state sovereignty and ideas about the leadership role of the WHO in a crowded global health governance context.

## Background

In 1948, the World Health Organization (WHO) was established as a specialized agency of the United Nations (UN). Since then, the agency has played a key role in several health achievements, such as the Alma-Ata Declaration on primary health care (1978), the eradication of Smallpox (formally recognized in 1980), the adoption of the Framework Convention on Tobacco Control (FCTC) in 2003, the revision of the International Health Regulations (IHR) in 2005, and the establishment of standards in medical care and medical ethics among others [1, 2]. However, the nearly 70-year-old global health agency has been widely criticized for being slow to reform itself to be able to effectively and efficiently function in a crowded and shifting global governance context [3, 4].

The WHO reform efforts began in earnest two decades ago, with the aim of making it a more streamlined, effective, responsive, transparent, and accountable organization, committed to improve health outcomes [5]. The first formal call for reform came from within WHO in 1993 [6] with the aim of overcoming institutional inertia [7]. According to the WHO constitution, the organisation should primarily be financed through regular contributions from member countries (i.e. an assessed contribution (AC) relative to the country’s wealth and population). These contributions are known as “regular budget funds” (RBFs). In addition to RBFs, article 57 of the constitution allows WHO to accept voluntary contributions (VC) from donors whose funding conditions are consistent with the objectives and policies of WHO. These contributions by the UN, member states, private organisations, or philanthropies are collectively referred as “extra-budgetary funds” (EBFs) [8, 9].

As early as 1949, the WHO recognised the considerable need to supplement resources over and above the RBFs [10]. Accordingly, from 1951 onwards, the WHO became a beneficiary of the UN expanded programme of technical assistance and the special fund that supports UN specialised agencies. This expanded programme receives VCs from major donor countries, but the allocations are made by the UN Technical Assistance Board or by the Special Fund [11]. In the early 1980s, the WHA introduced a “zero-real growth policy” for the regular budget. This policy froze membership dues (i.e. ACs) in real dollar terms so that only inflation and exchange rates would influence membership assessment adjustments [12]. In 1993, the WHA voted for a more stringent budgetary policy, moving the organisation from “zero real growth” to “zero nominal growth” for ACs. The zero nominal growth policy decoupled ACs from inflation or currency adjustments [13]. This policy shift made the organisation increasingly reliant on EBFs to uphold its constitutional role as the directing and coordinating authority for international health work (Art.2 of the constitution) [14].

In the late 2000s, member states began to raise concerns about the WHO’s limited financial resources, which they argued was hampering the organization’s ability to deliver expected outputs and to appropriately respond to emerging health issues. In response to those concerns, in 2010, the Director-General (DG) of WHO convened an informal consultation on the future of WHO financing. This consultation went beyond financing issues and raised more fundamental questions about the role of WHO in a rapidly changing environment. This consultation situated financing reform in broader programmatic reforms [15, 16]. According to the WHO, three fundamental challenges drove the ongoing reform efforts. First, the organization found itself overcommitted and overextended in its global initiatives. Second, the organization was compelled to re-examine its role in global health governance in relation to the increased prominence of multiple global health actors. For example, the emergence of public private partnerships (PPPs) such as the Global Fund, the GAVI and the UNITAID, and non–state actors such as the Bill & Melinda Gates Foundation with their strong financial resource base led to new actors playing a key role in global health. Last, the organization needed to develop the capacity and culture to be able to respond to new global health emergencies with sufficient speed and agility [17]. Following the Ebola outbreak, the emergency capacities of the WHO were added as a fourth field of reform [18].

Some of the acute financing challenges facing the WHO include misalignment between programme budgets and member states financial commitments, unpredictability of financing, lack of transparency of financing, and efficiency in resource management, vulnerability due to just 20 contributors funding 75% of the programme budget, and inflexibility of financing [19]. Scholars have argued that the financial resources of the WHO are incommensurate with its mandate [20]. To put this point into perspective, the WHO’s budget for the biennium 2018–2019 hovers around $4.421 billion (USD), while the annual healthcare and social services budget of Quebec, a Canadian province in which we live is approximately $33 billion (USD). Scholars note that the WHO is required to function on a budget equal to that of the University hospital in Geneva [21], and less than the budget of many major hospitals in the United States [20].

Many global health scholars have argued that WHO’s reliance on voluntary and earmarked contributions creates a situation where external donors dictate the organization’s priorities and action agenda [20]. The precarious financial situation of the WHO has given rise to extensive dialogue in the scholarly literature. This dialogue is intimately tied to existential questions about the future of the WHO in global health governance. To date proposals for financial reform have not been consolidated and synthesized. In this paper, we review the literature with specific reference to financial reform of WHO, synthesize proposals and recommendations made by global health scholars, and analyze them in the context of the ongoing discourse on the role and future of the WHO [22].

## Methods

We conducted a narrative review of the literature on WHO reforms to identify and summarize proposals for financial reform. We also sought to identify arguments and evidence used to support these proposals. In general, a narrative review summarizes a body of literature while identifying key discourses, summarizing common conclusions, or mapping a field [23]. We included peer-reviewed scholarly literature published in English, those containing either proposals or recommendations pertaining to the financial reform of the WHO, and evidence to support such proposals and recommendations. Dissertations and conference papers were excluded.

The literature was collected in two phases. The initial search was conducted between May-Aug 2015, and included relevant articles published between 1993 and 2013, noting that the first formal call for reform came from within WHO in 1993 [6]. Databases searched included Google Scholar, Medline, Global Health Database, Global Health (Archive) and PubMed. Search terms used were: “World Health Organization reform”; “Reform of the World Health Organization”; “World Health Organization” (as heading), “reform” (as keyword) – combined by “and”; “World Health Organization” (as heading), “reorganization” (as keyword) – combined by “and”. This initial search provided a total of 1221 hits. After screening the titles and abstracts, we identified 88 articles pertaining to WHO reform in general, out of these 41 made a reference to financial reform specifically and were included for full review.

We updated this search in early 2018 to include literature published between 2013 and February 2018, using the same search strategy. After removing duplicates (*n* = 104), we screened a total of 372 titles. The full text of 27 articles was reviewed and 15 articles were included for analysis. Including the initial 41 articles, a total of 56 articles were analysed to identify 1) suggested weakness pertaining to WHO financing, 2) financing proposals, 3) arguments to support the proposals, and 4) evidence (if any) to support the arguments.

### Analysis

We developed three overarching themes based on the content analyzed in the included articles: 1) Predictability and Sustainability of Organisational Funding, 2) Improve Transparency and Accountability in (Financial) Governance, and 3) Organizational Function and Financial Autonomy. Each of the specific proposals are presented in Table 1.

**Table 1:** Thematic overview of funding proposals and stated rationale

Reform proposal (s) and/or recommendations	Key arguments for reform proposal (s)/ challenges/ risks
Theme 1: Predictability and Sustainability of Organizational Funding	
Set higher member state ACs [29, 34–36]; Double the WHO’s overall budget, with ACs comprising at least 50% of budget within 5 years [53] **Since the Biennium 2014–2015, WHO gained the power to approve the full budget, enabling strategic allocation of flexible resources [52]	Sustainable financing needed to address WHO’s capacity to respond and nation’s preparedness [54] Member states reluctant to increase respective ACs and support increased VCs over ACs [36, 39, 50, 51]
Replace zero-nominal growth of ACs with zero-real growth policy [36, 39]	Zero-nominal growth policy restricted WHO’s budget growth [13, 38, 39], increased WHO’s reliance on EBFs [40]
Attract new donors (foundations, emerging economies, private and commercial sector) to broaden funding source for WHO’s flexible and unearmarked funds [26, 28, 29]	Tensions between financing from non-state actors/ private sector and WHO’s autonomy [34]; ‘trading off the soul of WHO’ [28]
Establish Ethics Committee to oversee WHO engagement with non-state actors [36]	BRICS countries asked WHO to ensure supremacy of member states and manage conflict of interests [36]
Charge overhead to VCs [47]	Might risk losing donors to other organizations [34]
Practice ‘currency hedging’ to manage currency risks [27] Establish endowment fund, a multiyear financing framework, or use Robin Hood tax [27]	
Theme 2: Improve Transparency and Accountability in (Financial) Governance	
Increase transparency in disbursements of funds to WHO regional offices & disclose its utilization [26, 30, 34]	
Build strategies for WHO’s rigorous external evaluations [27]	
Establish independent governance committees on the lines of Independent Monitoring Board [56, 57]	Towards transparency enhancement mechanisms [56]
Establish ‘WHO financing dialogue’ making the opaque process of multilateral negotiations more open and transparent, involving an inclusive discussion [43, 44] **In 2013, the World Health Assembly resolution 66(8) established the Financing dialogue [43]	Member states skeptical over the procedures of establishing WHO financing dialogue [62]; BRICS countries support to financing dialogue [36]; financing dialogue is ‘smoke screen exercise’ without resolving Zero-nominal growth policy [44]
Theme 3: Organizational Function and Financial Autonomy	
Narrow focus and concentrate resources on lesser health issues [61, 63, 66, 70]	
Decentralized governance through smaller independent organizations to bring efficiency and optimal use of funds [46, 68]	
Create discretionary fund’ for global health emergencies [26]	
Convene regularly a new multi-stakeholder forum to address critical global health issues [28]	This reform was argued as a way to better align WHO’s income and work [29, 43]
Greater autonomy for technical function of WHO through protected and adequate budget, with flexibility over its allocation [72]	
Acquire greater independence from its largest donors in order to coordinate with Research and Development (R&D) actors [77]	
Outsourcing of key activities, thus leveraging the expertise of global health organizations beyond WHO [67, 71–73]	
Taxation of global resources and global activities to supplement WHO funding [68]	

#### Predictability and sustainability of organizational funding

It is consistently noted that WHO is underfunded [24–32]. Beginning in the 1980s the WHO has faced reductions in disbursements from member states [13, 33], for example, the ACs from member states decreased significantly from 46% in 1990 to 21% in 2016–2017 [19]. In 2015, only 25% of the WHO’s programme budget came from ACs. The WHO’s operations costs have increased, however, the ‘proportional levies’ it obtains from member states have remained nominally stable [26, 27]. One of the proposals to overcome this funding challenge suggests setting higher member state contributions [29, 34–36]. However, to date, member states have been reluctant to increase their respective ACs [29, 36, 37] and Dr. Chan herself had stated that convincing parliaments and public of the need to increase funding to WHO was ‘hard to sell’ [26].

Many global health scholars argued that member states adherence to the “zero-nominal growth policy” has restricted budget growth of the WHO [13, 38, 39], thus increasing the organisation’s reliance on EBFs [40] including voluntary funds from member states and external contributors, which, now make up the majority of the funding. Scholars and experts have proposed to replace the zero-nominal growth of ACs with a zero-real growth policy [36, 39]. Furthermore, concerns have been voiced over the imbalance between ACs and EBFs creating challenges for sustainability and predictability of the WHO operations [34, 36, 41]. As noted earlier, over the past decade ACs have remained stable nominally and showed only slight growth by 3% in the 2018–2019 biennium budget. During this time, the proportion of programme budget financed through VCs over ACs increased significantly. For example, VCs accounted for nearly 78% of WHO’s programme budget for 2018–2019 [42]. It is also noteworthy that most of the VCs made to the WHO are earmarked for programmes and priorities driven by donor preferences rather than identified by the organization itself [41, 43–46]. Some scholars have argued that this earmarking practice contributes to skewing global health priorities [26, 34, 47] and weakening WHO’s decision-making power [48], shifting control of what is ostensibly a multilateral institution to wealthy member states and donors [28, 49]. It has also been argued that the practice of bypassing the regular budget through extra-budgetary funding demonstrates a lack of confidence in the WHO [29, 30, 41].

Despite the noted arguments above, member states continue to support increased VCs over ACs. Mackey and Novotny noted that USA has advocated for increased voluntary contributions [39] and UK’s House of Lords Select Committee on Intergovernmental Organizations requested that the UK Government make more funds through VCs available to WHO for its core activities [50]. The BRICS (Brazil, Russia, India, China and South Africa) Health Ministers’ meeting in Beijing on 7 July 2011 included discussions on how to increase voluntary contributions from LMICs [51]. The Republic of China has stated that it is important to increase flexibility of voluntary contributions and earmarked funding [36].

Yet another proposal favouring WHO finances put forth by the WHO DG report published in 2011 called for attracting new donors including foundations, emerging economies and the private and commercial sector to broaden their base for WHO’s flexible and unearmarked funds [26, 28, 29], However, there were expressed tensions between financing arrangements that permit non-governmental contributions, particularly from the commercial sector, and the ability to maintain institutional autonomy [34]. Richter emphasized that broader-based support and more inclusiveness may result in ‘trading off the soul of WHO’ and can pose a risk ‘to WHO’s role as the highest authority in international public health’ [28]. In line with the suggestion to attract donor funds, at the 133rd EB session in May 2013, there was extensive debate on non-state actor participation, and concerns were expressed about multi-stakeholder participation [36]. In that debate, BRICS countries emphasized the need to maintain the supremacy of member states and transparency of WHO’s interactions with the non-state actors. Further, they also proposed establishment of an Ethics Committee to oversee WHO engagement with non-state actors [36].

It has been argued that to ensure sustainable funding to the WHO, member states need to see themselves as shareholders [52]. However, in 2011 during the Ad Hoc Advisory Committee on reforming the WHO speech, the DG Chan observed that member states do not behave as “shareholders” with a genuine stake in the organization’s success [52]. This comment was prompted by member states resistance to implementing higher mandatory AC’s coupled with the continued push for unfunded mandates. To achieve sustainable funding, in 2015, Lawrence Gostin proposed that the WHA should double the WHO’s overall budget, with mandatory dues from member states comprising at least 50% of the budget within 5 years to increase the current proportion of ACs (20%) as against the VCs (80%) [53]. According to Gostin, sustainable financing must address both the WHO’s capacity to respond to, as well as each nation’s preparedness for, health emergencies [54]. One sign of the enhanced autonomy of the WHO is the power it was granted by the WHA to approve the budget in its entirety regardless of whether the funds are ACs or VCs, enabling strategic allocation of flexible resources [52]. This decision was established in 2014.

Others have proposed more technical and inventive solutions to increase financing. Pang and Garrett recommended that the WHO should practice “currency hedging”, which refers to a range of measures taken to manage currency risks (also known as exchange rate risk or foreign exchange risk). They further argued that much of the financial instability of the WHO is due to the lack of such measures to protect against currency risks [27]. Financing innovations have also been proposed, such as “the establishment of an endowment fund, a multiyear financing framework, or the use of a Robin Hood tax, which reaps financing from miniscule taxation of very large currency transactions” [27]. In general, a majority of WHO’s administrative costs are financed using ACs and funds generated through a programme support cost levy on VCs. Stenson and Sterky noted that the Executive Board (EB) Working Group proposed higher overhead rates on VCs in order to better integrate programmes funded by EBFs into WHO’s regular programme budget [47], though it is recognised that this proposal might risk losing donors to other organisations [34]. Currently, the WHO has the authority to assess appropriate overhead rates up to 35%, for extra-budgetary programmes.

#### Improve transparency and accountability in (financial) governance

Accountability and transparency are key features of good governance addressed in the literature [55]. Many analysts identify the lack of transparency and accountability as a key weakness of the WHO in regard to its performance and financial spending. This problem is intertwined with concomitant challenges of obtaining ACs [41, 56] and the practice of earmarking funds for specific projects from donors who are suspicious of mismanagement [28, 41, 56–58].

The need for greater transparency began to emerge from within WHO in 1990’s. At the 51st WHA, then DG, Gro Brundtland, emphasised: “WHO can and must change. It must become more effective, more accountable, more transparent and more receptive to a changing world” [49]. Dr. Bruntland also sought increased cooperation with the private sector under the name of public -privates partnerships [28]. Some scholars have argued that while Bruntland succeeded in establishing these partnerships, she did not succeed in strengthening organizational efficiency [59, 60]. Later, in 2011, during the tenure of DG Chan, the WHO EB supported the proposal from member states and the DG, to improve financing and governance, to facilitate communication across the WHO, and to increase transparency and accountability [61].

Several proposals have been made to improve transparency and accountability at the WHO [22]. In 2011, scholars proposed that the WHO needed to be more transparent regarding its disbursements to regional offices [30], including fully disclosing regional budgets [26]. Others take this proposal further to suggest that the WHO should disclose how regional offices use funds to meet health objectives, with monitoring and benchmarks of success [26]. According to Sridhar and Gostin, “stakeholders demand clarity on how their resources will achieve improved health outcomes as they shift toward results-based financing and performance-based measures” [34]. In line with this proposal, Pang and Garrett recommend the WHO to have a “strategy built around rigorous, external evaluations that demonstrate the value of its activities” [27]. Of late, transparency of funding and implementation progress in relation to regional offices has improved with the launch of a program budget web portal that is open to the public [42].

Another proposal was to establish a “WHO financing dialogue” in order to enhance transparency. It was suggested that this dialogue would make the process of multilateral negotiations more open and transparent, involving an inclusive discussion (i.e. with state and non-state actors) of budget agreements for stable and predictable financing by the member states [43]. This proposition was framed as an “innovative and transparent approach to secure the required funds” [44], however, concerns were raised about the potential risks of WHO’s increased reliance on non-state actors [44]. While some member states expressed skepticism about this procedure [62], the BRICS’ health ministers stated that “…they welcomed the initiation of the financing dialogue based on priorities collectively set by WHO member states in a structured and transparent process” [36]. Subsequently, in 2013, the WHA 66 [8] established the ‘Financing Dialogue’ aiming to ensure a match between WHO’s results and deliverables as agreed in the Programme Budget and the resources available to finance them, with the ultimate objective of enhancing the quality and effectiveness of WHO’s work. During the dialogue process, the member states and other non-state actors agreed on “guiding principles of WHO financing including, alignment and flexibility, predictability, transparency, and reducing vulnerability” [43]. However, scholars like Van de Pas and Van Shaik suggest that the financing dialogue is a “smokescreen exercise” until the member countries resolve other pivotal issues such as the zero-nominal growth policy for the assessment of member states dues to the WHO [44].

Most recently, in the context of the Ebola epidemic, Checchi and colleagues highlighted that WHO lacks sufficient flexible funding to respond to health emergencies, and called for enhanced transparency and accountability of organisational financing and spending as well as performance assessments [56]. They proposed establishing an external independent governance committee for WHO, modelled on the independent monitoring board (IMB) of the Global Polio Eradication Initiative (GPEI). Others have also called for the establishment of such committees and transparency enhancement mechanisms for WHO [57].

#### Organizational function and financial autonomy

The last theme encompasses proposals for securing WHO core functions, the role of WHO in global health governance, and maintenance of its leadership and autonomy in relation to other stakeholders. Many scholars have emphasised that financial autonomy is essential for the WHO global health agenda setting including health emergencies [53, 63]. However, insufficient funding from the member states, paralleled with increased proportions of EBFs, has limited WHO’s autonomy and constrained the organisation’s capacity to lead and coordinate global health [64, 65]. Several proposals have been made for efficient management of WHO’s financial resources.

Scholars have reiterated that WHO is overstressed with too broad a mandate and set of responsibilities, and that there is urgent need for protection of WHO’s core functions [63, 66] and to narrow the organisational focus [67]. This issue was initially recognized in 1997, when the DG announced that “WHO would concentrate its resources on a smaller number of health issues, but there have been only half-hearted efforts towards the constitutional reform that would make such a change in priorities possible. And there has been no high level discussion of changes to the organization’s structure or processes” [68]. It has been pointed out that at the time Dr. Bruntland was appointed as the DG of WHO, technical cooperative activities accounted for a majority of WHO’s budget, but this money was poorly spent on those activities and there was little sustained improvement in the health of poorer countries. The big donor countries argued that WHO should instead focus on its normative activities [58]. Other scholars have also highlighted the need for WHO to narrow its focus [61, 67] for specified amount of time and resources [69]. For example, Collier proposed narrowing WHO’s focus to five core areas: health development, health security, strengthening health systems and institutions, generating evidence on health trends and determinants, and convening for better health [70].

One suggestion is for WHO to concentrate exclusively on setting standards - the so-called normative functions- and to leave direct involvement in technical matters, also known as technical cooperation—to other agencies [71, 72]. A more recent reform model proposes outsourcing of key activities, thus leveraging the expertise of global health organisations beyond WHO [67]. The authors of this propositions argue that the WHO should aim to out- source a number of its functions to other global agencies that are already leading the way such as the Global Fund and GAVI. This would allow the WHO to focus on a small number of core activities where it has comparative advantage and to coordinate or orchestrate the broader array of global health actors to take on other activities [73]. The Global Fund and GAVI, unlike the WHO, have no funding stream called ACs or even expected levels of contributions by the donors. They are funded through VCs. For example, the Global Fund relies heavily on replenishment as its funding mechanism and receives funds from its RED^1^ partnership [13] to fight against HIV/AIDS, Tuberculosis and Malaria.

Another proposal for the management of financial resources has favored decentralised governance of WHO. A report commissioned by the Swedish ministry of foreign affairs emphasised that “the best way to achieve international cooperation in health would be through a decentralized network” consisting of “small independent organizations set up for limited periods to perform specified functions” [68]. The report suggests that these entities would be overseen by an assembly of member states and the secretariat but have their own finances and boards. It is suggested that such a system, without a regional structure, would free up funds. On similar lines, there have been recommendations to shift WHO’s resources into individual countries, instead of headquarters in Geneva or regional offices [46]. Interestingly, such a proposal to redistribute the organization’s budget for field operations threatened huge budget cuts for some of WHO’s regional operations, such as 50% from its Southeast Asian office [74].

A proposal to uphold WHO’s leadership in shaping future global health agenda came from the WHO former DG Margaret Chan in 2011. She proposed to convene a regular multi-stakeholder forum: “a new forum that will bring together member states, global health funds, development banks, partnerships, nongovernmental organizations, civil society organizations, and the private sector to address issues critical to global health” [28]. This reform proposal was presented as a mechanism for improving global health governance without being a formal part of the governance of WHO. While describing the merits of the forum, Andrew Cassels argued that “the reform plan started as a way of better aligning WHO’s income and work, and not to weaken the voice of member states or undermine WHO’s governance” [29, 43]. The idea was abandoned in November 2013 as it lacked support from member states [28].

Scholars have also noted that member states need to ensure that WHO has the necessary resources to effectively build the foundations needed to support them in achieving universal health coverage (UHC) and to address complex and expanded global health needs, including access to essential medicines [75]. However, this would require establishment of institutional frameworks, and rules and procedures, to make decisions about collective needs and actions [76]. According to Moon, the WHO needs to acquire greater independence from its largest donors. She acknowledged that WHO needs to reinforce its financial and political independence in order to strengthen its ability to coordinate with research and development actors [77]. Greater autonomy for the technical function of WHO’s Secretariat was also warranted [72]. One way to achieve this would be protected and adequate budget with flexibility over its allocation [72].

There has been a recommendation for the DG to create a “discretionary fund” which will be used for programmes in case of emergencies [26]. This fund will prevent the DG from having to collect funds during emergencies. This recommendation is consistent with WHO’s article 58: A special fund to be used at the discretion of the Board shall be established to meet emergencies and unforeseen contingencies. After the Ebola outbreak, in 2015, the WHA established the Contingency Fund for Emergencies (CFE) that finances WHO Health Emergency Programme, but the CFE is yet to obtain adequate resources [18]. In addition to RBFs and EBFs, other sources to fund WHO have been suggested including “new forms of taxation of global resources (such as oceanic sea beds, the air, or the genetic diversity in natural resources) and global activities (such as foreign trade, research, and intellectual property rights)” [68]. This proposal follows the UNITAID model, the international drug purchasing facility largely financed by a levy or tax on airline tickets.

## Discussion

In this paper, we have highlighted numerous critiques waged against the WHO financing structure and spending practices. We have also provided a survey of corresponding proposals to remedy these purported ills. These proposals have been debated, and less contentious issues, such as convening a financing dialogue, approval of the entire programme budget, and establishment of a health emergency programme have received member states consensus. However, more contentious ideas such as convening a multi-stakeholders forum and reversal of the zero-nominal growth policy of WHO funding have yet to receive approval from member states (particularly rich donor countries). Many of the more contentious reform proposals hinge on the formidable task of proving the effectiveness, efficiency and trustworthiness of the WHO system, along with the colossal mission of aligning actors around a unified vision for global health in a multipolar world [78].

### Challenges of ongoing WHO financial reform

In general, WHO’s priority setting is rooted in the need for cooperation with and among individual member states, based on assessments of disease burden, capacity, and national priorities, and the collective will of the member states, as expressed in conventions, regulations, and recommendations reflected in resolutions of WHO governing bodies, such as WHA and EB. Conditioned by a persistent funding gap, the organization’s priorities are increasingly influenced by the (major) donor member states and non-state actors [79]. This often undermines the organization’s ability to attend to the needs of less influential and less powerful countries. For example, the WHO leadership has prioritized UHC, implementation of International Health Regulations, social determinants of health, equity in access to medicines and health technologies, non-communicable diseases, and health related sustainable development goals (SDG). In contrast, many large donors have given priority to infectious diseases, to the extent that the WHO’s polio programme accounted for more than 20% of the WHO programme budget in 2016–2017 [80]. In the Biennium 2018–19 the polio program continued to receive the highest proportion of WHO allocated funds (902.8 million USD). This tension extends to the origins of the WHO and conflicts over whether the organization should focus on more technical health problems and solutions or pursue a social agenda rooted in an equity and human rights agenda.

This tension persists in the financing of discrete projects over general institutional, untied funds. David Stuckler and colleagues have noted that the skew towards infectious diseases was substantially greater for the WHO extra-budgetary funds [81], and this has been a persistent pattern in recent years [82]. Earmarked extra budgetary funds can help meet specific needs and evolving development challenges but can also make the coordination and coherence of international cooperation more difficult and undermine the strategic and coherent allocation of resources. Earmarked resources are intrinsically unpredictable, which makes it difficult for the WHO to carry out integrated planning for core earmarked resources [83, 84]. For example, earmarked funding of WHO left the organization ill-equipped to respond to international health emergencies, such as recent Ebola outbreak [85, 86]. Although non-earmarked contributions have increased, the contemporary global health landscape is characterised by a lack of coordination among highly resourced state agencies and non-state actors with often divergent priorities.

Over the past decade, the Bill & Melinda Gates Foundation has been the largest private donor to WHO, whose major contributions had been earmarked for polio eradication through the Global Polio Eradication Initiative (GPEI). GPEI funds contribute greatly WHO human resources, primarily in Africa [87]. The WHO Country Offices rely heavily on GPEI funded staff and other health programmes utilize the infrastructure created through the initiative. For example, polio-funded staff on the ground have helped to address public health emergencies, from the Ebola outbreak in West Africa and the recent drought in the Horn of Africa to the devastating earthquake in Nepal [88]. Apart from the potential loss of staff, shutting down the GPEI could leave the WHO with a significant financial short-fall. Though polio eradication is clearly a global public good, many fear that if the GPEI winds down in 2019 as anticipated, there will be a massive spin-off effect for the WHO. This example typifies the financing challenges facing the WHO in an environment where discrete health issues are pursued through short-term initiatives.

The current dominance of earmarked contributions in effect uses the WHO to channel donor priorities. Donors then benefit from the legitimacy and credibility of a democratic institution while circumventing the very processes that underpin and establish its legitimacy and credibility, the point here being that the WHO finds itself in an ever-crowded governance context and this reality is often beyond the control of the WHO [78]. What the members of the WHO can do is channel state commitment to global health by ensuring that the WHO is a transparent, efficient, and accountable institution that generates and enacts collective decisions. The democratic legitimacy of the WHO is pulled between charges of institutional deficiencies on the one hand and lack of support and capacity on the other. These two dimensions are intimately intertwined and as such, reform seems to require efforts to enhance the trustworthiness of the institution while also requiring greater trust and financial commitment by member states.

### Opportunities of ongoing WHO financial reform

The financial reform process has achieved key milestones such as establishing a financing dialogue in 2013. The financing dialogue has resulted in the establishment of guiding principles of WHO financing that include alignment and flexibility, predictability, transparency, and reducing vulnerability. The predictability of financing has improved over the past decade. For example, the level of predictability was 70% at the start of the biennium 2014–2015, compared with 62% for 2012–2013 and 52% for 2010–2011 [89]. The WHO Programme Budget Portal was established to enhance transparency and accountability [90]. Similarly, in 2015, the WHO accountability framework of 2006 was revised to ensure accountability and transparency of the general programme of work (GPW) and programme budget (PB) [91]. The implementation of this framework may enhance the credibility of the organization leading to greater trust from member states, and garner political will for increased financial commitments in the form of greater assessed contributions.

In strict monetary terms, the zero-nominal growth policy remains a critical barrier to enhanced institutional finances. The report of the Ebola Interim Assessment Panel highlighted that “the longstanding policy of zero nominal growth policy for assessed contributions has dangerously eroded the purchasing power of WHO’s resources, further diminishing the organization’s emergency capacity” [86]. The resistance to changing this policy points to two intersecting factors that are rooted in the political economy of state sovereignty.

First, the WHO must establish its presence as a trustworthy leader in the global health space. Although a number of member states were in favour of increasing assessed contributions, the Sixty- eighth WHA decided to maintain the ‘zero nominal growth policy’ [86]. The reasons cited include lack of political will and financial commitment of member states especially by the rich donor countries as they found inefficiency, lack of transparency, and minimal accountability within the organization [92]. For example, the delegation of the Netherlands, a major donor country which has a zero nominal growth policy for UN organizations, supported a freeze on assessed contributions to the WHO’s programme budget for 2016–2017 [92]. In addition, the economic interests [93] of rich member states also dampened support for the WHO. The United States, the largest donor for the UN and the WHO, has repeatedly opposed WHO taking any action which might run counter to the interests of transnational corporations. The US has opposed the Code on the Marketing of Breastmilk Substitutes, WHO’s rational use of medicines initiative and its ethical criteria for drug marketing, and yet to ratify the Framework Convention on Tobacco Control [13, 94]. Advancing the interests of corporations by the member states adversely effects WHO’s unbiased decision making and the means to refrain from ‘conflicts of interest’. For example, the sugar industry in the US demanded that Congress end funding to the WHO unless the WHO scrapped guidelines that sugar should account for no more than 10% of a healthy diet [95]. Similarly, the pharmaceutical corporations, who are also generous funders for presidential elections, value shareholders’ demand for profits over affordable access to essential medicines and vaccines [96].

The WHO continues to struggle in delivering its constitutional mandate amidst competing donor priorities. This situation has created a vicious cycle of mistrust, leading to a lack of commitment by member states to increase (assessed) contributions to the WHO, which then leads to the limited ability of the WHO to appropriately address major global health challenges, which itself erodes the place of the WHO as a leader in global health governance. Interestingly, the emerging economies such as the BRICS have formally committed to strengthen and legitimise the WHO as the coordinating authority in global health through the principle of multilateralism. These member states have been actively engaged in WHO reform process and striving to bridge the widened gap between assessed and voluntary contributions. For example, for year 2018, China is the largest contributor of ACs with 16.56 million USD, followed by Brazil with 5.91 million USD, Russian Federation with 4.45 million USD and India with 0.67 million USD [97].

Second, perhaps implicit in critiques waged against the ability of the WHO to function as global health leader and in the persistent reluctance to invest in the WHO, is an existential conundrum. The nature of multilateralism is that support is tied to a vision of the ‘global’, notions of the potential and possibility of cooperation and collective action. Over the past five years we have seen a dramatic reorientation of the global political order marked by an insular politics (e.g. Brexit, Trump’s America-First rhetoric) prompted in part by fears of terror, escalated geopolitical tensions, and populist leanings. The emergence of a politics of withdrawal from international cooperation does not capture the complexity of a global sphere that is also marked by greater international trade and greater social trans-border connectivity. However, a financing dialogue propelled by technical proposals masks a deeper reluctance of states to trust and thus invest in global, rather than international institutions. A core budget, although agreed upon by member states, does give extensive autonomy and authority to the bureaucracy of the WHO. Barnett and Finnmore, provide a convincing analysis of international institutions and argue that there is reason to see entities, such as the WHO, as “ontologically independent actors” [98]. Following this logic, we contend that, in part, the reason why states are reticent to invest in the core budget of the WHO, is that by doing so they are partly relinquishing their state sovereignty, a dominant norm shaping international politics. This relinquishing of state sovereignty using resources generated through state government is a perplexing philosophical challenge that underpins the WHO financing discourse but is not given explicit attention. It is this second point that requires greater attention in the research and scholarship on WHO financing arrangements.

The proposals that we have highlighted in our review demonstrate that there is no simple solution to WHO financing reform. Reform is necessarily tied to deeply entrenched political and economic conditions, as well as ideological positions about the relationship between individual governments and inter-governmental agencies. This review provides an important overview of challenges that underlie any reform proposals and it is hoped that our analysis can provide important background for future dialogue on WHO financing reform.

## Conclusions

Improving the transparency, flexibility, predictability of WHO’s financing and upholding organisational autonomy are at the centre of WHO financing reform. With the on-going WHO financing reform, the level of predictability and transparency has improved, but the alignment and flexibility of member states contributions is yet a major challenge. The WHO financing dialogue, as an innovative and transparent approach, continues to enhance the efficiency and effectiveness of the resource mobilisation and spending. Despite the progress, WHO’s heavy reliance on donors for funding major programmes significantly limits the organisational autonomy and global leadership. Increased ACs for biennial budget remains a pressing challenge for the organisation as a leader in global health agenda setting and implementer of global plans, such as the sustainable development goals.

## Footnotes

1 The RED Partnership was founded in 2006 by Bono and Bobby Shriver to harness the power of people and companies to help fight AIDS. The Global Fund uses 100% of RED partnership money without any overhead charges to finance HIV/AIDS programs.
